# Effects of admixture in native and invasive populations of *Lythrum salicaria*

**DOI:** 10.1007/s10530-018-1707-2

**Published:** 2018-03-21

**Authors:** Jun Shi, Mirka Macel, Katja Tielbörger, Koen J. F. Verhoeven

**Affiliations:** 1 Institute of Ecology and Evolution, Plant Ecology Group, University of Tübingen, 72076 Tübingen, Germany; 2 Ningbo Academy of Agricultural Sciences, Ningbo, 315040 China; 3 Department of Plant Science, Radboud University Nijmegen, P.O. Box 9010, 6500 NL Nijmegen, Netherlands; 4 Department of Terrestrial Ecology, Netherlands Institute of Ecology (NIOO-KNAW), Droevendaalsesteeg 10, 6708 PB Wageningen, Netherlands

**Keywords:** Admixture, Biological invasions, Heterosis, Inbreeding depression, Phenotypic plasticity, Purple loosestrife

## Abstract

Intraspecific hybridization between diverged populations can enhance fitness via various genetic mechanisms. The benefits of such admixture have been proposed to be particularly relevant in biological invasions, when invasive populations originating from different source populations are found sympatrically. However, it remains poorly understood if admixture is an important contributor to plant invasive success and how admixture effects compare between invasive and native ranges. Here, we used experimental crosses in *Lythrum salicaria*, a species with well-established history of multiple introductions to Eastern North America, to quantify and compare admixture effects in native European and invasive North American populations. We observed heterosis in between-population crosses both in native and invasive ranges. However, invasive-range heterosis was restricted to crosses between two different Eastern and Western invasion fronts, whereas heterosis was absent in geographically distant crosses within a single large invasion front. Our results suggest that multiple introductions have led to already-admixed invasion fronts, such that experimental crosses do not further increase performance, but that contact between different invasion fronts further enhances fitness after admixture. Thus, intra-continental movement of invasive plants in their introduced range has the potential to boost invasiveness even in well-established and successfully spreading invasive species.

**Electronic supplementary material:**

The online version of this article (10.1007/s10530-018-1707-2) contains supplementary material, which is available to authorized users.

## Introduction

Biological invasions are a global problem and we still do not know why some introduced plants become invasive whereas others do not (Kolar and Lodge 2001). Some widely studied and partially supported hypotheses emphasize the role of biotic interactions to explain increased vigor of invasive species, e.g. the enemy release hypothesis (ERH) and the evolution of increased competitive ability hypothesis (EICA) (Keane and Crawley 2002; Joshi and Vrieling 2005; Puliafico et al. 2008; Felker-Quinn et al. 2013). However, less attention has been paid to alternative evolutionary genetic processes such as the consequences of admixture (Ellstrand and Schierenbeck 2000; Verhoeven et al. 2011; Bock et al. 2015; van Kleunen et al. 2015). Admixture has potential enhancing effects for population success, for instance by increasing genetic variation, creating novel genotypes and masking effects of fixed deleterious recessive mutations leading to heterosis (Rieseberg et al. 2003; Seehausen 2004; Verhoeven et al. 2011; Rius and Darling 2014). One underlying reason for heterosis after admixture is that individual populations often suffer from some level of inbreeding depression (Angeloni et al. 2011), especially when populations are small. Inbreeding depression is in part caused by accumulation and expression of deleterious recessive mutations and leads to plant fitness decrease (Charlesworth and Willis 2009). Admixture between diverged populations can alleviate the negative effects of inbreeding depression by masking the effects of deleterious mutations that were fixed in individual populations, contributing to heterosis (Xiao et al. 1995). An alternative effect of admixture, observed in crosses between very distant populations, can be outbreeding depression, due to dilution of local adaptation and disruption of co-adapted gene complexes that have diverged over evolutionary time (Waser and Price 1989; Verhoeven et al. 2011).

In early invasions, population bottlenecks often cause loss of genetic variation. Continued inbreeding in small founder populations can speed up purging of deleterious mutations (Facon et al. 2011) but this may not always be efficient enough to eliminate the negative effects of inbreeding (Frankham et al. 2001), suggesting that masking of deleterious mutations by admixture is beneficial during early invasions. Thus, when invasive species are introduced via multiple introductions from different native source populations, these diverged populations can readily meet and admix in the invasive range (Dlugosch and Parker 2008; Facon et al. 2008; Verhoeven et al. 2011) and this admixture may cause strong heterosis and boost invasive plant fitness in the early invasive stages (Drake 2006). Although heterosis is a transient effect, it can substantially boost the chance for initial population establishment (Drake 2006). Furthermore, admixture might continue to promote invasiveness in established invaders when new genotypes are introduced from the native range (van Kleunen et al. 2015).

The benefits of admixture apply also to native range populations, although it has been argued that local adaptation in native range populations reduces the benefits of admixture due to dilution of locally adapted gene pools (Verhoeven et al. 2011). This presumably plays a smaller role in invasive populations that have not yet evolved strong local adaptation. Thus, the balance between the costs and benefits of population admixture plays out differently in native populations compared to early invaders, with stronger selection for admixture in early invaders because there is less cost associated with diluting locally adapted gene pools. When early invaders are introduced from multiple source populations, it can therefore be hypothesized that selection for admixture leads to admixed invasion fronts (Ellstrand and Schierenbeck 2006); whereas source populations from the native area maintain a higher degree of differentiation (Verhoeven et al. 2011). This prediction can be tested empirically by evaluating offspring performance from within and between-population crosses and comparing these between native and invasive populations. Despite much interest in the role of admixture and evolutionary change in invaders few such studies exist (e.g. Wolfe et al. 2007; Rius and Darling 2014; van Kleunen et al. 2015), and consequently our insight in the role of admixture in biological invasions remains limited.

Here, we use *Lythrum salicaria* (Purple loosestrife) as a model species to study the effects of admixture in invasions. *L. salicaria* is a perennial, wetland-associated herbaceous plant. Well-established populations can survive in dry habitat for many years (Blossey and Schroeder 1995), but usually *L. salicaria* occurs along rivers, lakes, or other wet habitats. *L. salicaria*’s relatively wide tolerance to different water stress levels in the recruitment phase may be a potential factor for its wide spreading in North America during last 200 years (Keddy and Ellis 1985). In the 1800 s, *L. salicaria* was initially introduced from native European populations into the east coast of North America (Thompson et al. 1987). Multiple introductions occurred along the east coast and both historical and genetic evidence indicate that this led to admixture between different native source populations (Thompson et al. 1987; Houghton-Thompson et al. 2005; Chun et al. 2009). After the 1850 s, *L. salicaria* was recognized as a potential horticultural and landscape plant. It may be in this way that the species was first introduced into the Great Lakes region (Midwest) and also into the Pacific Northwest were small and widely scattered populations have been present since the early twentieth century (Stuckey 1980; Edwards 2012). Explosive invasive westward spread occurred since the 1930 s from the east coast introduction areas, possibly involving further admixture between local Midwestern populations and Westward spreading populations (Edwards 2012). Likewise, inland spread also occurred from the Northwestern *L. salicaria* sites into e.g. Idaho, Utah, Wyoming and northern California (Edwards 2012).

To investigate the effects of experimental admixture, we used *L. salicaria* from nine native European populations and nine invasive North American populations. Within both ranges we performed experimental crosses within and between populations at different geographic distances and we evaluated F1 offspring under two environmental treatments, wet and drought soil conditions, because the expression of heterosis can be environment-dependent (Velasco et al. 1987). We tested the hypotheses that (1) in native populations, admixture increases *L. salicaria* performance with increasing parental distance; and (2) in the invasive range, experimental admixture does not lead to improved performance because mixing between multiple introductions has already resulted in admixed populations (Thompson et al. 1987; Houghton-Thompson et al. 2005; Chun et al. 2009); crosses among such admixed populations are not expected to result in further heterosis.

## Materials and methods

### Study species

*Lythrum salicaria* L. (Purple loosestrife; Lythraceae) is an erect, wetland herbaceous perennial (Thompson et al. 1987). It is self-incompatible and flowers are pollinated by long-tongued insects (Agren 1996). It is heterostylous and each plant produces one of three morph-specific patterns: long-, mid- or short-styled morph (Waites and Agren 2004). The trimorphic system in *L. salicaria* avoids self-pollination (Colautti et al. 2010a, b) as legitimate pollination requires the deposition on the stigma of pollen from anthers of equivalent height, which are found only between different flower style lengths (Oneil 1992; Eckert et al. 1996; Waites and Agren 2004). In the invasive range, multiple introductions to Eastern North America are thought to have given rise to an admixed invasion front (Houghton-Thompson et al. 2005; Chun et al. 2009) in which adaptive population differentiation has evolved along the front’s northern edge in response to climatic factors (Colautti and Barrett 2013).

### Plant materials and experimental crosses

Seeds of *L. salicaria* were collected from populations in two ranges, Europe and North America, between 2004 and 2012; several of the seed provenances used had been included also in previous *L. salicaria* studies (e.g. Chun et al. 2009; Moloney et al. 2009). Within each range, seeds were used from three geographically distant regions and within each region we used three populations (Table 1). Distances between regions were larger in the North American range than in the European range, and in North America distances between populations within a region were larger in western regions than in eastern regions (specifically, population distances within regions were 3–15 km in the European samples; 4–15 km in the New Jersey samples; 25–31 km in the Idaho samples; and 113–200 km in the Iowa samples). From previous work it is known that genetic variation and population differentiation are higher in Europe than in North America, and within North America are higher in eastern than in western regions (Chun et al. 2009). The differences in the spatial scale of our population sampling reflect these differences in genetic variation, with spatially broader sampling in regions with lower variation and/or differentiation to capture sufficient variation also in areas of reduced variation. Distances between populations in both native and invasive areas were larger than distances that are usually covered by natural honey bee pollinators (Beekman and Ratnieks 2000 and references therein) and also larger than typical *Lythrum salicaria* seed dispersal (mostly < 10 m; Thompson et al. 1987). In October 2012, seeds from 12 to 15 mother plants per population were sown in petri-dishes with water in a greenhouse (NIOO-KNAW, Wageningen, Netherlands) with 16 h light, 8 h dark and a constant 20 °C. Two weeks later, one seedling per mother plant was transplanted into 1.5 L pots filled with steamed commercial potting soil. In total, 228 seedlings were planted in the same greenhouse as used for the germination with the same conditions. Around 50 days after transplantation, three types of pollinations were made: (1) between plants within a single population (intrapopulation crosses), (2) between plants from different populations in the same region (interpopulation crosses) and (3) between plants from different populations in different regions within a range (inter-region crosses). Crosses were made within the native and invasive ranges, but not between the two ranges. For each of the 18 populations, the 12–15 plants grown per population were used both as seed parent and as pollen donor for all cross types. Due to incompatibility within the style morphs [tristylous mating system (Eckert et al. 1996)], not all seed parents could be used for all cross types. In each population, we designated six individuals as seed parents and each of these seed parents was used for all three cross types, receiving pollen from randomly selected and compatible pollen donors from the respective populations and regions. This design efficiently controls for differences between seed parents when evaluating the effects of cross type on offspring. Moreover, maternal effects do not influence long-term growth and phenology in *L. salicaria* populations (Montague et al. 2008). In some populations one or two of the six seed parents did not produce F1 seeds for all three cross types. In such cases, F1 seeds were added from other seed parents who contributed to only one or two cross types. In our study, populations were identified by the identity of the seed parent (not pollen donor). In April 2013, the seed capsules from each plant were harvested and stored at 4 °C.

**Table 1:** Geographic locations of *Lythrum salicaria* populations used in this study

Origin	Region	Population	Latitude	Longitude	Mean annual temp. (°C)	Annual precipitation (mm)
Europe (native)						
	Tübingen	Hagelloch (TH)*	N48°33′	E09°01′	11.0^a^	592.6^a^
		Unterjesingen (TU)*	N48°31′	E08°59′		
		Reusten (TR)*	N48°33′	E08°56′		
	Potsdam	Geltow (PG)	N52°22′	E12°57′	10.8^a^	553.8^a^
		Ferch (PF)	N52°20′	E12°55′		
		Golmer Luch (PL)	N52°24′	E12°57′		
	Wageningen	Ewijk (NE)	N51°52′	E05°45′	11.3^b^	1002.0^c^
		Nijmegen (NN)	N51°51′	E05°53′		
		Wageningen (NW)	N51°58′	E05°40′		
North America (invasive)						
	Idaho	Star Idaho (ISI)	N43°42′	W116°29′	9.9^d^	377.3^d^
		Middleton (IML)	N43°25′	W116°22′		
		Boise River (IBR)	N43°36′	W116°11′		
	Iowa	Boone Folks (IABF)*	N42°17′	W93°56′	9.34^d^	929^d^
		Little South Storm Lake (IALS)*	N42°38′	W95°14′		
		Manly (IAMA)*	N43°16′	W93°07′		
	New Jersey	New Jersey Site 1 (NJS1)	N40°59′	W74°44′	11.4^d^	1218.2^d^
		New Jersey Site 3 (NJS3)	N41°05′	W74°43′		
		New Jersey Site 4 (NJS4)	N41°07′	W74°43′		

*Same populations as used in Chun et al. (2009)

^a^http://www.wetterkontor.de/ (2014–2015, temperature and precipitation of Potsdam and Tübingen (Stuttgart) regions)

^b^https://weerstatistieken.nl/ (2014–2015, temperature of Wageningen (De Bilt) region)

^c^http://historie.neerslagkaart.nl/ (2014–2015, precipitation of Wageningen region based on source KNMI)

^d^ https://www.currentresults.com/Weather/US/weather-averages-index.php

### Greenhouse experiment

Potting soil was mixed with 20% pumice. 10 cm × 10 cm × 10 cm pots were filled with 650 g planting soil. Per pot, 10 g Osmocote slow-release fertilizer pellets were added (considered as a relatively high nutrient level; Bastlova et al. 2004). During the experiment, pots were placed individually in plastic containers to hold excess water. Greenhouse conditions were kept at 16 h light and 8 h dark with a 21 °C/16 °C temperature (day/night) during the experiment.

Seeds were germinated in petri-dishes. After 2 weeks, all germinated seedlings from each family and cross type were selected randomly and transplanted into the pots and placed on greenhouse benches. The experiment followed a replicated randomized block design. Each replicate block contained 108 plants: two origins (native/invasive) ×  three regions per origin × three populations per region × three cross types × two soil treatments (drought/wet) × 1 replicate. Due to limited greenhouse space the experiment was performed in two successive time blocks: three of six replicate blocks were included in the first Time Block which started from 1th March 2014 to end of April; another three of six replicates were included in the second Time Block from 10th May 2014 to 10th July. Several seedlings (3.5% of total) did not establish within 1 week after transplantation and were removed from the experiment. More than half of these were from the native Tübingen region. We observed that in this region seedling failure was not higher in intra-population crosses than in other cross types, suggesting that not inbreeding depression but an other, unknown, factor caused seedling failure. Drought treatment was applied by adding 300 mL water to each pot every 3 days; in the wet treatment, pots were maintained continuously in a shallow layer of water (2 cm deep) during the entire experiment which simulated a normal, optimal growing environment (Keddy and Ellis 1985).

After 6 weeks of growth in the greenhouse, plants were harvested. Plant height was measured (cm) from the surface of the soil to the shoot tip. The biggest leaf of each plant was selected visually and its width and length (cm) were measured. A proxy of leaf area was calculated by multiplying the width and length of the biggest leaf. The diameter of the main stem (mm) was measured 2 cm above the soil surface. The number of side branches (> 2 cm long) from the main stem was determined. Shoots and roots were harvested after thorough rinsing to remove soil from the roots and shoot and root dry weight were determined after oven drying for at least 72 h at 65 °C. In *L. salicaria*, vegetative size was previously shown to correlate linearly and positively with total dry biomass, vegetative dry biomass, and total fruit production (Colautti et al. 2010a, b), indicating that biomass can be used as a proxy for plant fitness.

### Statistical analysis

Statistical analyses were conducted using SAS 9.2 (SAS Institute, Cary, NC). We used linear mixed models (PROC MIXED) to test effects of cross type, soil treatment, origin and all their 2- and 3-way interactions on plant traits (fixed effects). Because the experiment was divided over two time periods, we stratified the data by including time block and its interaction with soil treatment as additional fixed effects. The model accounted for random effects of replicate block (nested within time block), population (nested within origin) and the 2- and 3-way interactions of population with soil treatment and cross type. Because strong heteroscedasticity was observed associated with the soil treatments, we fitted unequal-variances mixed models that accounted for different variances for the two treatment levels. We fitted a priori contrasts to evaluate the effect of cross type within each combination of origin and soil treatment. A variant of this model was fitted including only within-population crosses to test for phenotypic plasticity differences (in response to the drought-wet treatment) between native and invasive plant populations.

Additionally, in order to single out the effects of admixture within and between different invasion fronts, we performed an additional analysis that separated the North American data into two parts: (1) dataset one excluded offspring from crosses involving an Idaho parent, and thus only included offspring from cross types involving New Jersey and/or Iowa regions (the Midwest/Eastern invasion front); (2) dataset two only included offspring from crosses that involved at least one parent from Idaho (representing the Northwestern invasion front). A similar mixed model as described above was fitted to these two data sets, including cross type, soil treatment, the cross type * soil treatment interaction, time block and the time block * soil treatment interaction as fixed effects, while accounting for random effects of replicate block (nested within time block), population and the population * soil treatment and population * cross type interactions.

## Results

### Origin and treatment effects on plant growth

**Fig. 1:**
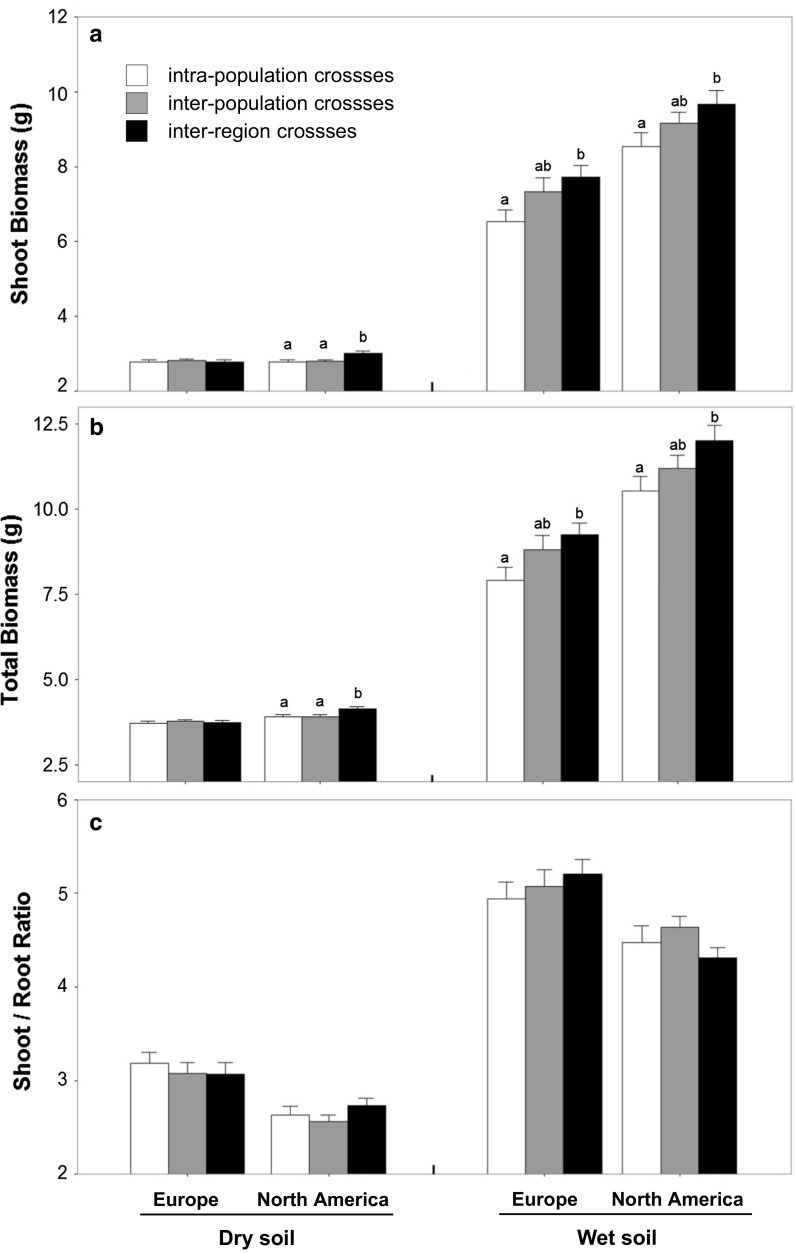
Comparison of cross type effects in native (Europe) and invasive (North America) plants on shoot biomass, total plant biomass and shoot/root biomass ratio, as determined in F1 wet soil conditions and dry soil conditions. Statistical differences between cross types within each origin and soil treatment were assessed by linear contrasts and are indicated by different letters. The soil treatment effect was significant (*p* < 0.0001) for all three traits

**Fig. 2:**
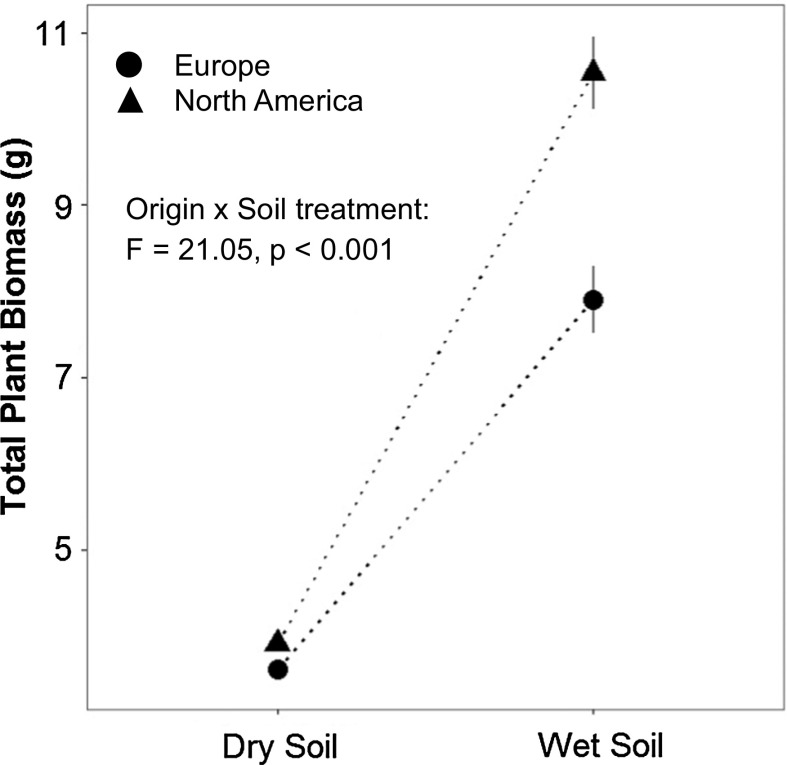
Effect of soil treatment on total plant biomass in native and invasive *L. salicaria* populations. Statistical testing used the same mixed model as described for Table 2, but using data from intra-population crosses only

Invasive plants had significantly higher shoot biomass, higher total plant biomass, and lower shoot/root biomass ratio than native plants (Fig. 1; Table 2). The dry soil treatment reduced shoot biomass, total plant biomass and shoot/root biomass ratio compared to wet soil conditions (Fig. 1; Table 2). Significant interactions between origin and soil treatment were found for shoot and total plant biomass (Table 2). We subsequently tested for differences in plasticity to soil wet-dry variation between native and invasive populations by including only within-population crosses in the analysis. These analyses confirmed stronger responses to the experimental soil treatments in invasive populations: plants from both ranges showed similar total plant biomass under drought conditions, but invasive plants benefitted much more than native plants did from optimal (wet) growing conditions (Fig. 2). Most of the other phenotypic traits also exhibited greater plastic responses to soil treatment in invasive plants than in the natives (Fig. S1; Table S1).

**Table 2:** Mixed model analysis results for shoot biomass, total plant biomass and shoot/root biomass ratio

Variable	*df*	Shoot mass	Total mass	Shoot/root
Origin	1, 16	55.57***	78.43***	25.22***
Cross type	2, 32	8.22**	8.06**	0.10
Soil treatment	1, 16	1787.38***	1649.75***	739.37***
Time block	1, 4	39.68**	62.59**	0.42
Cross type × origin	2, 32	0.15	0.26	0.36
Cross type × soil treatment	2, 32	5.72**	5.76**	0.95
Origin × soil treatment	1, 16	54.91***	62.40***	0.79
Cross type × origin × soil treatment	2, 32	0.12	0.02	2.31
Soil treatment × time block	1, 510	68.65***	74.08***	2.30

The table presents F values and significance for fixed effects; see main text for random effects that are accounted for in the models. *p* values that remain significant after false discovery rate correction for multiple testing (at table-wide FDR = 0.05) are italicized. Degrees of freedom (*df*) are specified for numerator and denominator respectively

*p < 0.05; **p < 0.01 and ***p < 0.001 respectively

### Cross type effects on plant performance

Cross type effects varied among the individual populations (Fig. S2) but significant overall effects of cross type were found on shoot biomass and total plant biomass, with heterosis increasing with distance between populations (Fig. 1; Table 2). The effect of cross type on biomass was significantly influenced by soil treatment (significant cross type * soil treatment interaction), but the cross type effect was not significantly different between the two origins (Table 2).In invasive populations, F1 offspring from inter-region crosses showed better plant performance than offspring from intrapopulation crosses, which was illustrated by higher shoot and total plant biomass in both the dry soil and wet soil treatments (Fig. 1a, b). In native populations, inter-region crossed offspring also showed better plant performance than intrapopulation crossed offspring with significantly higher shoot and total plant biomass in wet soil treatment, but not in dry soil condition (Fig. 1a, b). There was no significant effect of cross type on the shoot/root biomass ratio in either the invasive or the native populations under both soil treatments (Fig. 1c).

### Cross type effects within and between different invasive routes

The effect of experimental crosses on plant performance was contingent on the different invasion fronts that have been documented for *L. salicaria* in North America.

**Fig. 3:**
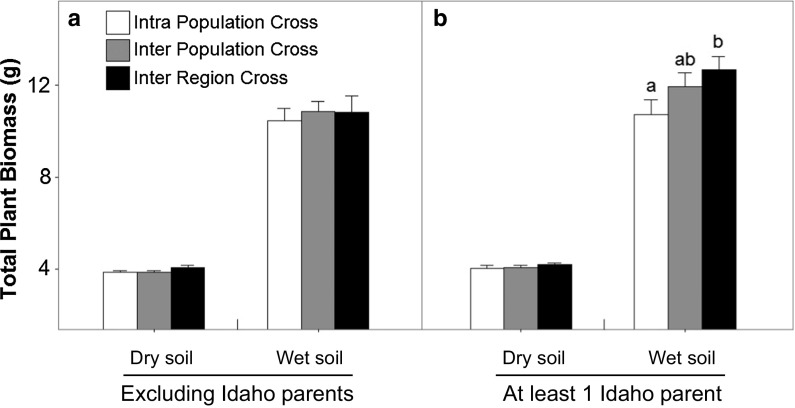
Comparison of cross type effects on total plant biomass based on two different subsets of crosses that either include at least one parent from the Western invasion front (‘Idaho’) or that include only parents from the Eastern/Midwest invasion front (‘No Idaho’), in dry and wet soil treatments. Statistical differences between cross types within each origin and soil treatment were assessed by linear contrasts and are indicated by different letters. The soil treatment effect was significant (*p* < 0.0001) in both data subsets

When analyzing the North American plants including only east coast and Midwest populations (from the same North-eastern invasion route, thus excluding crosses involving Idaho populations that derived from the Western invasion route), the effect of cross type on plant biomass disappeared (Fig. 3a; Table 3). In contrast, and although no overall significant effect was observed across both soil treatments (Table 3), the a priori contrast analysis of the crosses that involved at least one parent from Idaho indicated a significantly higher total biomass in inter-region crosses compared to intrapopulation crosses under wet soil conditions (Fig. 3b). Combined with the results of the overall analysis including all crosses (Fig. 1b), this indicates that heterosis was observed in the invasive range in crosses between the Eastern/Midwestern invasion fronts and the Western invasion front, but not in crosses within these two fronts.

**Table 3:** Mixed model results for total plant biomass based on two different subsets of crosses that either include at least one parent from the Western invasion front (‘Idaho’) or that include only parents from the Eastern/Midwest invasion front (‘No Idaho’)

Variable	Idaho		No Idaho	
	*df*	F value	*df*	F value
Cross type	2, 4	3.65	2, 10	0.84
Soil treatment	1, 8	578.06***	1, 5	324.36***
Time block	1, 4	23.12**	1, 4	45.24**
Soil treatment × cross type	2, 106	2.46	2, 148	0.28
Soil treatment × time block	1, 106	31.82***	1, 148	47.50***

The table presents F values and significance for fixed effects; see main text for random effects that are accounted for in the models. *p* values that remain significant after false discovery rate correction for multiple testing (at table-wide FDR = 0.05) are italicized. Degrees of freedom (*df*) are specified for numerator and denominator respectively

*p < 0.05, **p < 0.01 and ***p < 0.001 respectively

## Discussion

We predicted that experimental crosses between populations and regions express heterosis in the native range (because native-range populations are differentiated resulting in some inbreeding depression), but not in the invasive range (because multiple historical introductions and selection for admixture has led to already-admixed invasion fronts). Contrary to prediction, our results showed heterosis in between-population crosses both in native and invasive populations of *L. salicaria*. However, heterosis in invasive plants was limited to crosses involving parents from two different Eastern and Western invasive routes. The species has a documented history of multiple introductions to Eastern North America with likely post-introduction admixture (Chun et al. 2009). We therefore propose that our result reflects an already-mixed Eastern/Midwestern invasion front, in which experimental admixture does not provide an additional heterosis benefit anymore. Strikingly, such a heterosis effect is still possible when plants from different and invasion fronts mix. This highlights the potential of admixture to enhance invader fitness, also long after initial invader establishment. It also emphasizes the importance of efforts to avoid contact between different invasion fronts within an invaded continent in order to reduce the invasive success of exotic plants.

Even when our observed absence of heterosis in crosses within the Eastern/Midwestern invasion front suggest a lack of genetic differentiation between populations within this area, it is well known that invasive plant populations can rapidly evolve local adaptation to variation in environmental conditions in the new range (Bock et al. 2015). In fact, such adaptive differentiation has been demonstrated in *L. salicaria* populations in response to climatic factors along the northern edge of this Eastern/Midwestern invasion front (Colautti and Barrett 2013). Adaptive differentiation between populations may be less pronounced along the east–west cline of invasive spread, and if such adaptive differentiation occurred it was not reflected in heterosis effects in our crosses. Possibly, adaptive differentiation evolves more rapidly along latitudinal than longitudinal clines due to stronger climatic differences.

In our study, invasive plants were larger than native plants. The bigger size of invasive *L. salicaria* is consistent with previous observations in this species (Blossey and Notzold 1995; Bastlova and Kvet 2002; Mal and Lovett-Doust 2005; Chun et al. 2009; Joshi and Tielborger 2012; Joshi et al. 2014) and for invasive species in general (Gallagher et al. 2015). Several non-exclusive hypotheses can explain this pattern. For instance, the EICA hypothesis predicts that invasive plants escape from natural enemies in their introduced range and subsequently evolve to allocate fewer resources to defenses and more to growth and reproduction (Blossey and Notzold 1995), and previous studies with similar populations has confirmed this hypothesis for *Lythrum* (Joshi et al. 2014). Admixture effects, such as demonstrated in our study, can be an additional contributor to increased vigor.

In native-range plants, admixture between different populations boosted plant performance with increased parental distances. This might reflect the effects of deleterious mutations that accumulated in natural populations due to local inbreeding. Molecular markers in a previous study illustrated a significant pattern of isolation by distance (between populations from Potsdam, Tübingen and Switzerland regions) in *L. salicaria* (Chun et al. 2009). Additionally, both the Tübingen and Wageningen population sizes are small (< 500 flowering individuals, pers. observations J. S. and K.J. F. V.). Small populations usually suffer more from inbreeding depression (Angeloni et al. 2011). The observed heterosis in experimental crosses between native populations may therefore be largely from lifting inbreeding depression (Charlesworth and Willis 2009). Outbreeding depression may also occur if the parental distance was large enough (Edmands 1999). However, we did not detect evidence for outbreeding depression the between native range populations, which were separated ~ 600 km maximally.

Admixture may be more beneficial for invasive than for native populations, because invasive populations can benefit more from novel gene combinations in order to adapt to novel selection pressures (Ellstrand and Schierenbeck 2006) or because the negative effect of disrupting locally adapted genomes is less important in recent invaders (Verhoeven et al. 2011). Our results indicate that when the species is introduced to its invasive range through multiple introductions from different source populations, admixture is expected to lead to a fitness boost via heterosis. In invasive populations, we observed an admixture effect only in crosses between different western and an Eastern/Midwestern invasion fronts. Over extensive geographic areas within the Eastern/Midwestern invasion front, experimental crosses showed little evidence of heterosis, despite well-documented multiple introductions. This is consistent with the idea that this large *L. salicaria* invasion front consists of already-admixed populations (Thompson et al. 1987; Houghton-Thompson et al. 2005; Chun et al. 2009), such that further experimental admixture has little added effect on plant fitness. In North America, short-distance seed dispersal and pollen exchange are mainly driven by wind and insect pollination respectively (Grout et al. 1997; Thompson et al. 1987). Long-distance seed transport and/or seed spread occurred when seeds were embedded in mud adhering to wildlife, humans and vehicles, and trade as an ornamental plant (Mullin 1998; Mitich 1999; Stuckey 1980). These factors will promote admixture within the invasive Eastern/Midwestern front. However, our data showed that further heterosis was expressed after admixture between populations from different invasion fronts, when plants from these regions were crossed with invasive plants from a western US invasion route, suggesting the isolation between these two invasive fronts exists. Our results suggest that the further admixture between different invasion fronts may contribute to invasive success. However, in our study we only used one region in the western invasion front and it will be relevant to confirm the generality of our findings using multiple regions of both invasion fronts.

Our results suggest that admixture could contribute to plant performance in early invasion stages (as suggested by heterosis in inter-region crosses in native *L. salicaria*) but also much later in the process of invasive spread, as illustrated by heterosis in inter-region crosses between different invasion fronts. However, effects of admixture for invasive success were not overwhelming, because F1 offspring from inter-region crosses of native *L. salicaria* were still smaller than offspring from intrapopulation crosses of invasive *L. salicaria*. Possibly additional factors have contributed to the evolution of vigorous invasive plants, for instance the evolution of increased competitive ability (EICA) after escaping from specialized natural enemies (Joshi et al. 2014).

Surprisingly, the effects of heterosis in *L. salicaria* were greater in optimal wet soil conditions than in more stressful drought conditions. However, most previous studies showed that inbred individuals are often more sensitive to environmental stress than outbred individuals (Fox and Reed 2011). This would predict more beneficial admixture effects under stressful conditions. However, our results may indicate that inbreeding depression sometimes is expressed more strongly in beneficial environments. Alternatively, it is possible that heterosis in our study was not associated with the lifting of inbreeding depression due to deleterious recessive mutations but with other mechanisms, such as the environment-dependent expression of overdominance or epistasis (Fethi et al. 2011).

In our study, we observed that *L. salicaria* from both native and invasive ranges showed similar total biomass under drought conditions, but invasive plants benefitted more than native plants did from wet (optimal) growing conditions. This difference in phenotypic plasticity between native and invasive populations suggests a ‘master of some’ strategy of the invasive plants (Richards et al. 2006), where invasive success is determined more by the capacity for rapid opportunistic growth when conditions are favorable than by the capacity to maintain fitness under a range of harsh conditions. This result is consistent with observations in other species (Davidson et al. 2011). A previous study also showed that invasive *L. salicaria* has phenotypic plasticity in both vegetative and reproductive traits (Mal and Lovett-Doust 2005). At high nutrient levels (as in our study), both shoot and root biomass exhibited greater phenotypic plasticity to increased water levels in invasive *L. salicaria* compared to native genotypes (Chun et al. 2007). Thus, our results supported previous findings of higher plasticity in invasive *L. salicaria*. In general, phenotypic plasticity is seen as an important contributor in colonization in environmentally diverse areas and plays a role in invasions (Williams et al. 1995; Kaufman and Smouse 2001; Maron et al. 2004).

In conclusion, our finding of clear heterosis effects after experimental crosses between native range populations but not between invasive range populations from a single large invasion front, despite well-documented multiple introductions, is consistent with the idea that the invasion front consists of already well-admixed populations. Admixed invasion fronts are predicted to arise given the enhancing effects of heterosis and other admixture advantages for invasive spread. Strikingly, however, our finding of clear heterosis in crosses between different invasion fronts shows that further boosts to invasiveness in this well-established invader are expected when different invasion fronts meet. To minimize the ecological and economic impact of plant invasions, this emphasizes that it is important to think about avoiding not only new introductions from the native range, but also to avoid movement of propagules within the invasive range in order to prevent admixture between populations from different invasion fronts.

## Data, code and materials

Data and statistical analysis code that are used in this article can be accessed at the Dryad digital repository: 10.5061/dryad.tr69js8.

## Electronic supplementary material

Below is the link to the electronic supplementary material.

Supplementary material 1 (DOCX 465 kb)

Supplementary material 2 (JPEG 488 kb)
