# Supplementary material for: Effects of admixture in native and invasive populations of Lythrum salicaria
Source: Biol Invasions. 2018 Mar 21;20(9):2381–93. doi: 10.1007/s10530-018-1707-2 (PMC6417435; doi:10.1007/s10530-018-1707-2)

**Supplementary information**

Table S1. Results from mixed model analysis based on intra population crosses for additional phenotypic traits measured in our study in *L. salicaria* as dependent variable. Time Block and Origin were set as fixed factors. Population and Experimental Block were set as random factors and were nested in Origin and Time Block respectively. Random factors were not shown here. Table entries are F-values. P-values that remain significant after false discovery rate correction (significance at level of 0.05 and corrected across all P-values in this table) are underlined. Shoot = Shoot Biomass, Root = Root Biomass, Total = Total Plant Biomass, Ratio = Shoot Biomass / Root Biomass, Leaf = Biggest Leaf Area, Diameter = Diameter of Main Stem, Branch= Number of Branches from Main Stem.

| Variable | Shoot | Root | Total | Ratio | Height | Leaf | Diameter | Branch |
| --- | --- | --- | --- | --- | --- | --- | --- | --- |
| Soil | 429.71*** | 144.58*** | 413.94*** | 138.5*** | 378.98*** | 318.31*** | 731.16*** | 285.16*** |
| Origin | 17.56*** | 31.71*** | 24.11*** | 9.32** | 0.94 | 6.69* | 13.52** | 1.59 |
| Origin × Soil | 19.32*** | 14.84** | 21.05*** | 0.11 | 7.9* | 0.85 | 9.45** | 6.39* |
| Time Block | 9.87* | 7.29 | 14.23* | 0.88 | 14.05* | 0.87 | 6.03 | 20.11* |
| Time Block × Soil | 12.17*** | 24.31*** | 15.56*** | 6.26* | 4.27* | 15.88*** | 14.48*** | 8.23** |

*, ** and *** indicate *P* < 0.05, *P* <0.01 and *P* < 0.001 respectively.

**Figure S1**. Phenotypic traits in F1 of of intrapopulation crosses of native and invasive *L. salicaria* in dry and wet soil treatments. Solid circles and triangles are native and invasive plants respectively. *, ** and *** indicate the significant interaction between origin and soil treatment at the levels of *P* < 0.05, *P* < 0.01 and *P* < 0.001 respectively, based on the statistical model in Table S1.


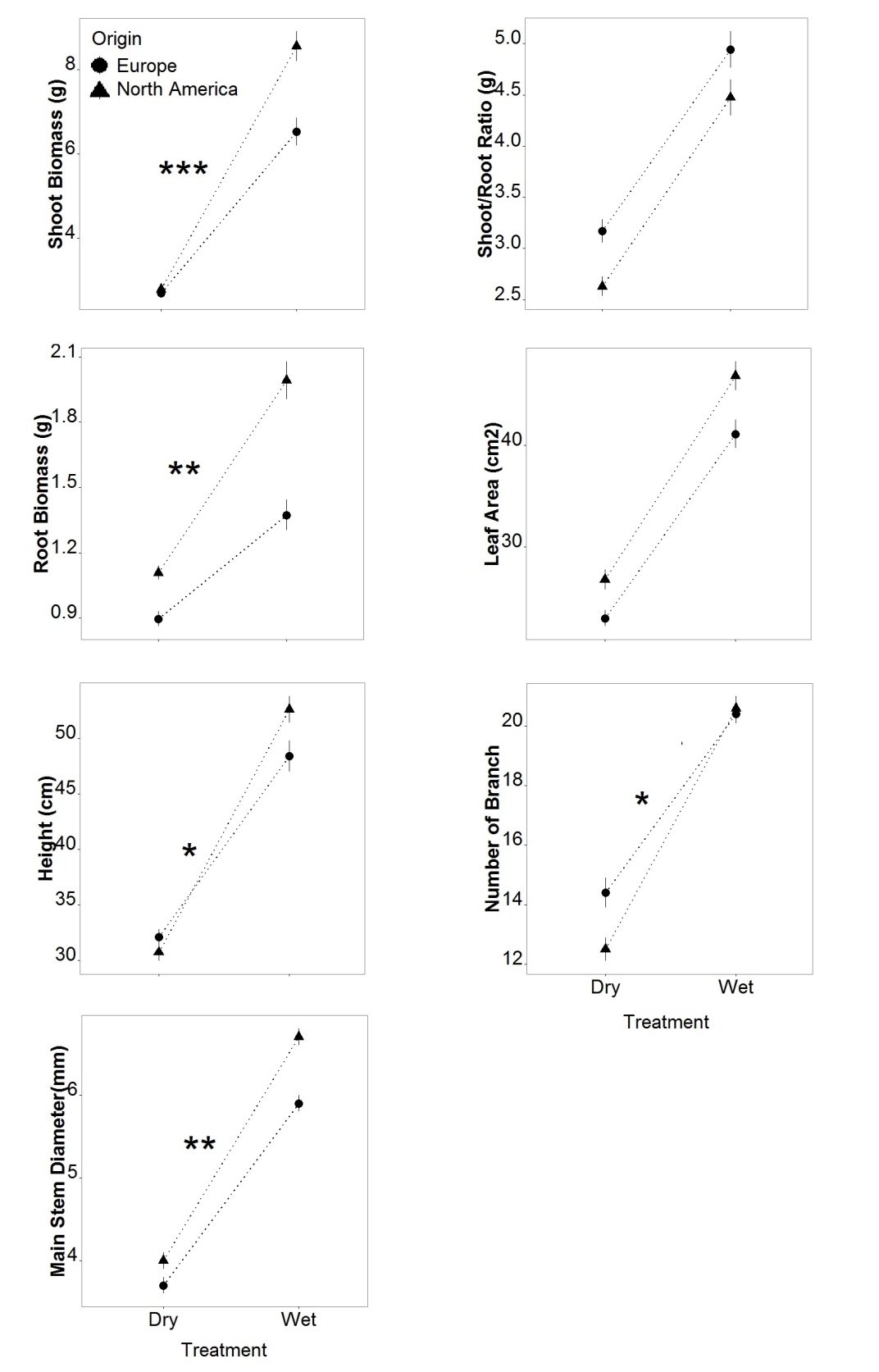


**Figure S2.** Effects of admixture on total plant biomass in each native (panels a-i) and invasive (panels j-r) *L. salicaria* population under dry and wet soil treatments. Population abbreviations and cross type color coding are as in Table 1 and Fig. 1 (white: intra-population; grey: inter-population; black: inter-region crosses).


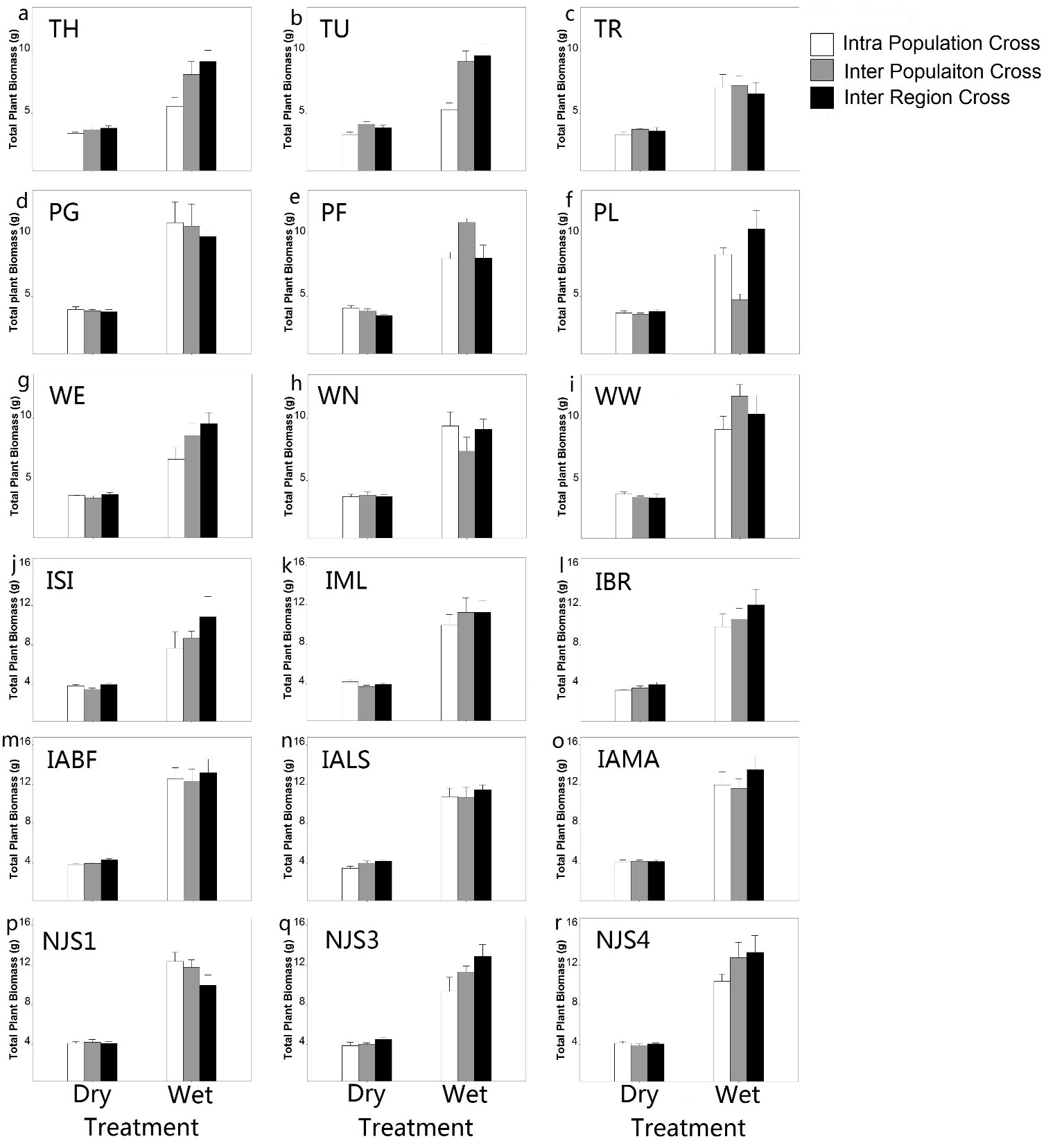

Supplement: Supplementary file 1 — Supplementary material 1 (DOCX 465 kb) [file 10530_2018_1707_MOESM1_ESM.docx]
