# Supplementary figures and images for: Effects of admixture in native and invasive populations of Lythrum salicaria
Source: Biol Invasions. 2018 Mar 21;20(9):2381–93. doi: 10.1007/s10530-018-1707-2 (PMC6417435; doi:10.1007/s10530-018-1707-2)

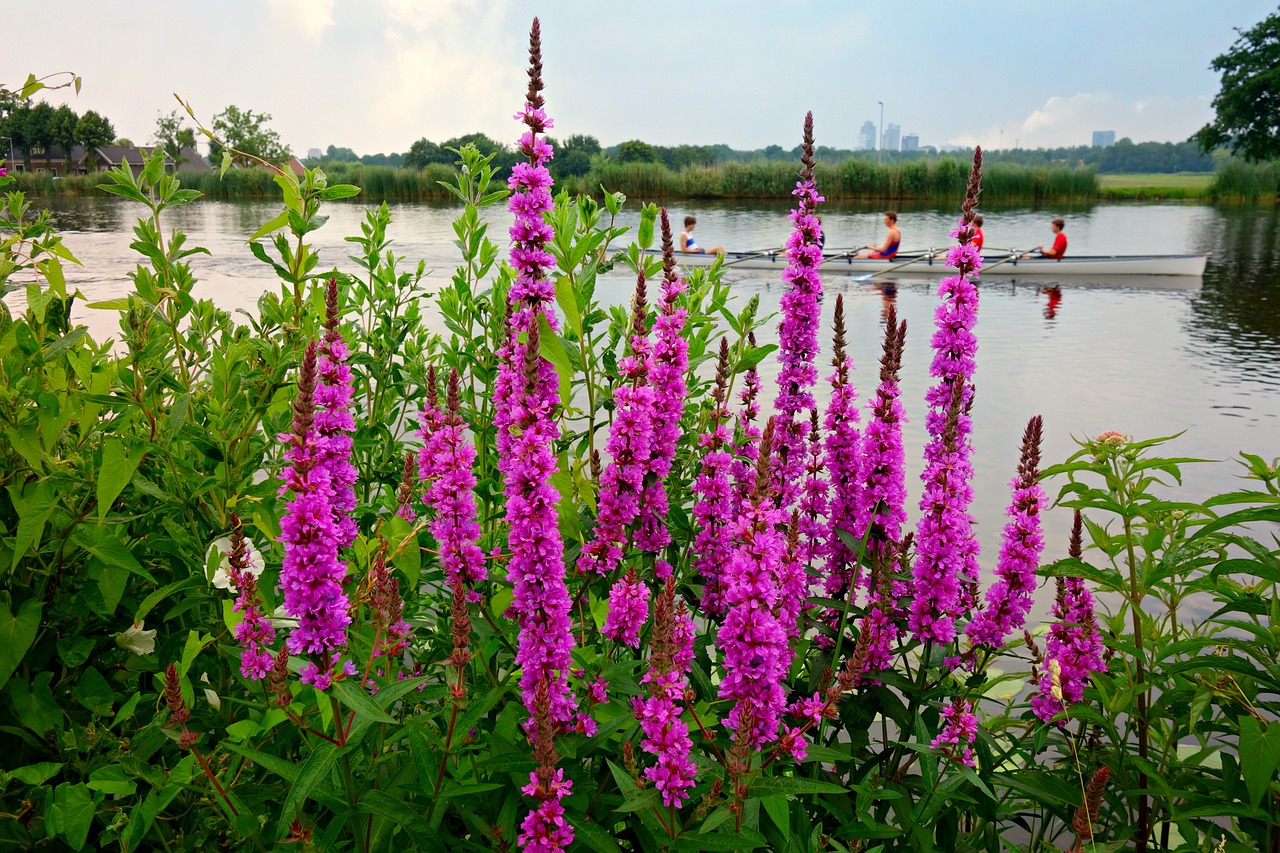

Supplement: Supplementary file 2 — Supplementary material 2 (JPEG 488 kb) [file 10530_2018_1707_MOESM2_ESM.jpg]
